# Longitudinal evaluation of innate immune responses to three doses of CoronaVac vaccine

**DOI:** 10.3389/fimmu.2023.1277831

**Published:** 2023-10-02

**Authors:** Cheng Cao, Junfeng Jiang, Min Liu, Yaping Dai, Tianzhi Chang, Tuo Ji, Fang Gong

**Affiliations:** ^1^ Department of Laboratory Medicine, Jiangnan University Medical Center, Wuxi, Jiangsu, China; ^2^ Department of Laboratory Medicine, Changzhou Jintan First People’s Hospital, Changzhou, Jiangsu, China; ^3^ Wuxi School of Medicine, Jiangnan University, Wuxi, Jiangsu, China; ^4^ Department of Laboratory Medicine, Affiliated Hospital of Jiangnan University, Wuxi, Jiangsu, China; ^5^ Department of Laboratory Medicine, The Fifth People’s Hospital of Wuxi Affiliated Hospital of Jiangnan University, Wuxi, Jiangsu, China

**Keywords:** CoronaVac, vaccination, innate immunity, monocytes, natural killer cells, IFN-γ

## Abstract

The adaptive immune responses induced by inactivated COVID-19 vaccine has been extensively studied. However, few studies have analyzed the impact of COVID-19 vaccination on innate immune cells. Here in this study, we recruited 62 healthcare workers who received three doses of CoronaVac vaccine and longitudinally profiled the alterations of peripheral monocytes and NK cells during vaccination. The results showed that both the monocyte and NK cell subsets distribution were altered, although the frequencies of the total monocyte and NK cells remained stable during the vaccination. Additionally, we found that both the 2^nd^ and 3^rd^ dose of CoronaVac vaccination elicited robust IFN-γ-producing NK cell response. Our data provided necessary insights on innate immune responses in the context of three homologous CoronaVac dose vaccination, and supplied immunological basis for the future design of inactivated vaccines against SARS-CoV-2 or other viruses.

## Introduction

Highly contagious severe acute respiratory syndrome coronavirus 2 (SARS-CoV-2) caused global pandemics of coronavirus disease 2019 (COVID-19) that lead to a huge impact on human physical and mental health (1, 2). COVID-19 vaccination, such as mRNA, adenoviral-based and inactivated vaccines, has allowed the containment of SARS-CoV-2 infection and mortality (3). Owing to their widespread use, numerous scholars have conducted extensive research on the adaptive immune responses mediated by T and B cells after vaccination (4–10). By contrast, little is known about the innate immune responses affected by vaccination.

The innate immune system constitutes the first-line responder to virus invasion, in which NK cells and monocytes are two important innate immunocytes. Using a systems vaccinology approach, Arunachalam et al. reported that, following a second dose of Pfizer/BioNTech BNT162b2 mRNA vaccination, a notably expansion of CD14^+^ monocytes in a group of 56 healthy volunteers (11). Moreover, the monocyte-related gene signatures were found to be associated with neutralizing antibody titers (11). Consistently, it was further discovered the convergence of monocyte and natural killer (NK) cell subsets during vaccine-induced responses. CD16^+^ NK cells, CD56^high^ NK cells, and non-classical monocytes inversely correlated for the magnitude of neutralizing antibody responses (12). In addition, multiple evidences have indicated that regulating NK cells may contribute to improve vaccine efficiency (13). However, a detailed map of the monocytes and NK cell landscape elicited by CoronaVac inactivated vaccine, following the second and third vaccine booster, has not yet been established.

In this research, we evaluated the CoronaVac vaccine-mediated long-term effects on innate immune responses by analyzing monocyte and natural killer subsets in peripheral blood, as well as IFN-γ production after SARS-CoV-2 spike peptide pool stimulation. Analyses were performed at pre-vaccination baseline (T1), 1 week post the primary dose (T2), 2 weeks post the 2^nd^ dose (T3), 6-8 months post the 2^nd^ dose (T4), and 2 weeks post the 3^rd^ booster dose in a group of healthcare workers who had never been SARS-CoV-2-infected.

## Materials and methods

### Study population

In this study, we longitudinally collected the peripheral blood of 62 healthcare workers from Affiliated Hospital of Jiangnan University. According to the national vaccination scheme, all these participants received 2-3 doses (0.5 mL per dose) of the inactivated CoronaVac vaccine (Sinovac Life Sciences, Beijing, China) through intramuscular regimens from December 2020 to November 2021 (**Table S1**). EDTA blood was collected from all of the subjects at the following five time points: pre-vaccination baseline (T1), 1 week post the primary dose (T2), 2 weeks post the 2^nd^ dose (T3), 6-8 months post the 2^nd^ dose (T4), and 2 weeks post the 3^rd^ booster dose (T5). These healthy subjects ranged in age from 28 to 67 (median age 47), including 33 males and 30 females. All the individuals have no SARS-CoV-2 exposure or other virus infection, with no autoimmune disease, immunodeficiency disease, allergic diseases or any other diseases during the vaccination. This study was approved by the medical ethical committee of the Affiliated Hospital of Jiangnan University (LS2021004). All individuals included in the study provided written informed consent.

### Preparation and storage of peripheral blood mononuclear cells

The peripheral blood samples were collected in EDTA-2K tubes (BD Biosciences) and processed within 4 hours. As previously reported (14), blood samples were diluted with phosphate buffer saline (PBS) (1:1), and then slowly added on the prepared Ficoll-Hypaque (GE Healthcare Life Sciences) for density gradient centrifugation (450 g, 25 min, 20°C, without brake). PBMC layers were carefully collected and washed twice with PBS. After centrifugation, the washed PBMCs were resuspended in the cell cryopreservation solution (fetal bovine serum with 10% dimethyl sulfoxide), and total living cells were counted. Aliquots of 2.5 x 10^6^ cells were first transferred to a freezing container (Corning Incorporated) at -80°C overnight, then cryopreserved in liquid nitrogen until further use.

### Immunophenotyping using flow cytometry

Surface staining and multiparametric flow cytometry analysis were performed under the same conditions. Briefly, cells were washed with FACS buffer (PBS with 2% FBS) and incubated with Fc-receptor block (Mitenyi Biotec) to block non-specific staining. Cells were then stained with a mixture of monoclonal antibodies, including CD3-AF532 (Clone: UCHT1), CD19-Super Bright 436 (Clone: HLB19), CD16-eFlour 450 (Clone: CB16), CD56-BV711 (Clone: HCD56), HLA-DR-APC/Fire 750 (Clone: L243), CD14-PE-Cy5 (Clone: 61D3), CD38-PerCP-eFluor 710 (Clone: HB7), CD161-Brilliant Violet 785 (Clone: HP-3G10) (**Table S2**). After incubation in the dark at 4°C for 30 minutes, cells were resuspended in FACS buffer and kept on ice until acquisition by Cytek™ Northern Lights. Dead cells were routinely excluded from the analysis by staining with 7-AAD (eBioscience).

Monocytes were identified as CD3^-^CD19^-^CD14^+^ cells. Monocyte subpopulations were analyzed by CD14 and CD16 (12, 15): classical (CD14^+^/CD16^-^), intermediate (CD14^+^/CD16^+^), and non-classical (CD14^low^/CD16^+^) monocytes. The expression of HLA-DR on CD14^+^ total monocytes was determined using the median fluorescence intensity (MFI) for protein expression. For NK cells, we selected CD3^-^CD19^-^ live lymphocyte population according to 7-AAD. NK cells were divided into 6 subsets according to the intensity of CD56 and CD16 (16–18): (1) CD56^bright^CD16^neg^; (2) CD56^bright^CD16^dim/bright^; (3) CD56^dim^CD16^neg^; (4) CD56^dim^CD16^dim^; (5) CD56^dim^CD16^bright^; (6) CD56^neg^CD16^bright^.

### IFN-γ secretion assay

The levels of IFN-γ secretion by NK cells were measured by the following procedures. Around 1×10^6^ cells/well thawed PBMCs were plated in U-bottom 96 well plate containing complete RPMI-1640 medium (Gibco) with 5% heat inactivated FBS (Gibco). Cells were cultured in medium alone (unstimulated) or stimulated with SARS-CoV-2 spike peptide pools (S1 + S2, 2 µg/mL respectively, SinoBiological) for 24h at 37°C and 5% CO_2_. After 24 hours of culture, Brefeldin A (1:1000, eBioscience) was added to the culture for an additional 4 hours. Next, cells were harvested and stained with a mixture of surface antibodies, including CD3-AF532, CD19-Super Bright 436, CD16-eFlour 450 and CD56-BV711. Fc-receptor block (Miltenyi Biotec) was added simultaneously to incubate in the dark at 4°C for 30 minutes. After the surface staining, cells were fixed and permeabilized using BD Cytofix/Cytoperm Fixation and Permeabilization Solution Kit, and stained with intracellular antibody IFN-γ-PE-Cy7 for 30 minutes at room temperature in the dark. Stained cells were resuspended in FACS buffer and flow cytometry was performed using a Cytek™ Northern Lights instrument.

### Statistical analysis

Data were analyzed using FlowJo 10.6.2. Statistical analyses were performed in GraphPad Prism 9.2.0 software. Mann-Whitney U test was used for the comparison of the frequencies of monocytes and NK cell subsets between five time points. Wilcoxon matched-pairs signed-ranks test was applied for paired comparisons of IFN-γ^+^ total NK and IFN-γ^+^ NK subsets frequencies between different time points. *P* values and all tests are two-sided. A *P* value of 0.05 was used to determine significance of differences. *P < 0.05; **P < 0.01; *** *P* < 0.001; **** *P* < 0.0001.

## Results

### Alteration of monocyte frequencies and subsets during CoronaVac vaccination

**Figure 1:**
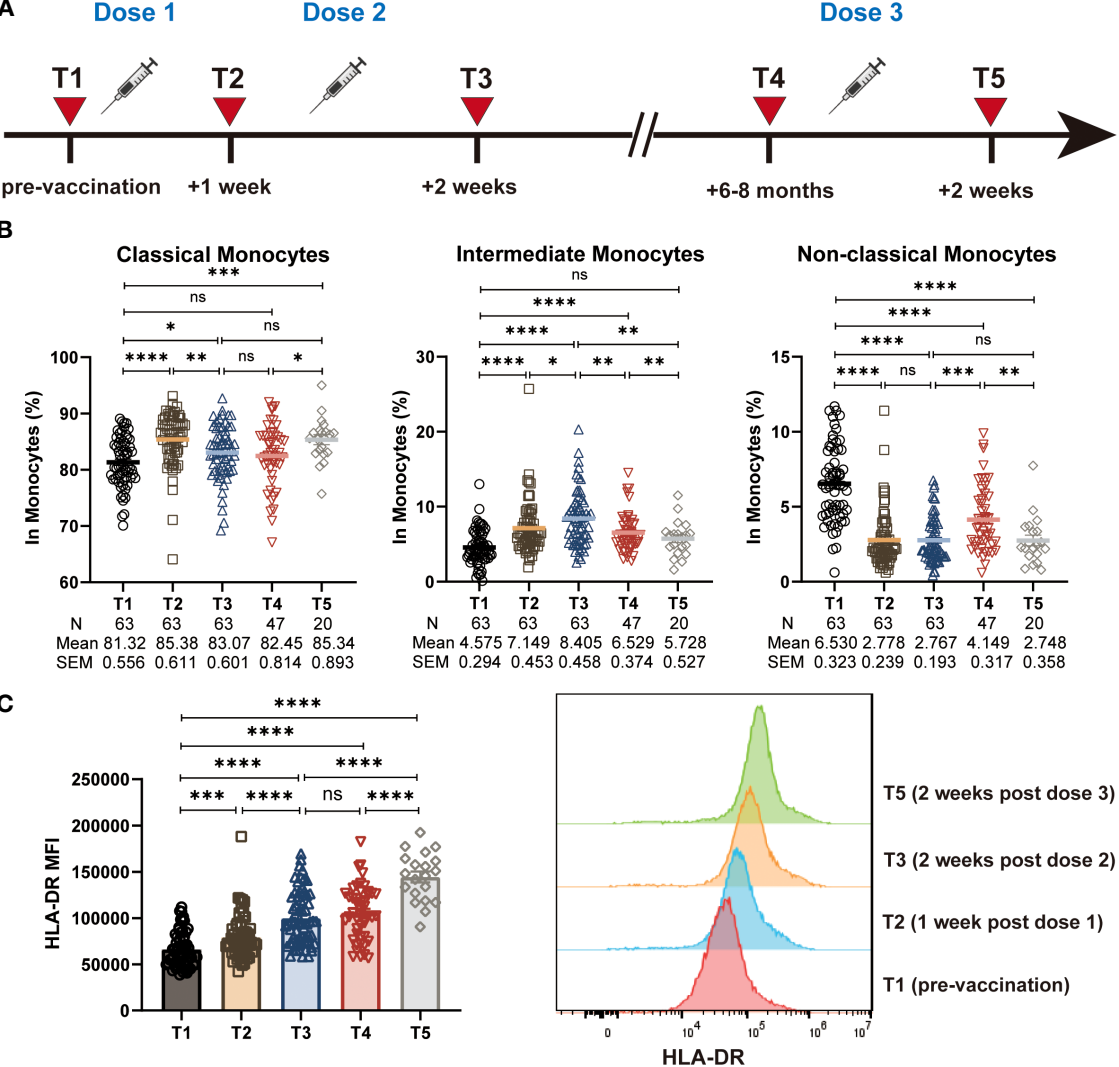
Characterization of monocyte frequencies and subsets during CoronaVac vaccination. **(A)** Study design. T1: pre-vaccination baseline, T2: 1 week post the primary dose, T3: 2 weeks post the 2^nd^ dose, T4: 6-8 months post the 2^nd^ dose, T5: 2 weeks post a 3^rd^ booster dose. **(B)** Statistical analysis of the frequency of polyclonal classical, intermediate and non-classical monocytes at five time points. **(C)** The median fluorescence intensity (MFI) of HLA-DR expression on total monocytes at five time points. Histogram showing the HLA-DR expression on total monocytes at T1, T2, T3, and T5. Each dot represents an individual subject. Bars in the figure represent the mean values with SEM. Mann-Whitney U test was used for the comparison between time points **(B, C)**. *, *P* < 0.05; **, *P* < 0.01; ***, *P* < 0.001; ****, *P* < 0.0001; ns, not significant.

In this study, we longitudinally followed 62 healthcare workers vaccinated with CoronaVac for over 300 days. This cohort has also been previously described the longitudinal magnitude of antibody response, as well as CD4^+^ and CD8^+^ T cell responses after vaccination (9, 19, 20). Blood samples were collected at the following five time points: pre-vaccination baseline (T1), 1 week post the primary dose (T2), 2 weeks post the 2^nd^ dose (T3), 6-8 months post the 2^nd^ dose (T4), and 2 weeks post a 3^rd^ booster dose (T5). 260 PBMC samples were collected at these five time points following three doses of CoronaVac vaccination (**Figure 1A**).

We first assess the effect of the CoronaVac vaccine on the frequency of the total monocyte population. However, different from the results from the ChAdOx1 nCoV-19 vaccine (21), we observed that the frequency of total monocytes (CD14^+^) remained stable during the vaccination (**Figure S1A**). Monocyte subpopulations can be identified as CD14^+^CD16^-^ classical monocytes, CD14^+^CD16^+^ intermediate monocytes and CD14^dim^CD16^+^ non-classical monocytes (**Figure S1B**). When evaluating the functional monocyte subpopulations, we found that the CoronaVac vaccine induced an increased frequency of CD14^+^CD16^+^ intermediate monocytes and CD14^+^CD16^-^ classical monocytes, together with a decreased frequency of CD14^dim^CD16^+^ non-classical monocytes (**Figure 1B**).

### Expression of antigen presentation molecule HLA-DR on monocytes during CoronaVac vaccination

Antigen presentation by APCs such as monocytes to activate effective T cell responses, which eventually leading to the production of antibodies or long-term immunological memory, is a crucial event in the vaccination process. There is evidence indicating that high levels of HLA-DR expression on the surface of monocytes associated with enhanced antigen presenting capacity and immune activation, whereas the low levels associated with immune suppression (22, 23). Therefore, we sought to evaluate the effect of the CoronaVac vaccination on antigen presentation capacity of monocytes by assessing the level of HLA-DR expression. Expression of HLA-DR on CD14^+^ total monocyte was significantly increased at all time points after vaccination (T2, T3, T4, T5), compared with the pre-vaccination baseline (T1). Of note, the expression of HLA-DR remained the relatively high level 6-8 months post the 2nd dose of vaccine (T4), with a continued increase post the 3rd vaccination (T5) (**Figure 1C**). In addition, we found that the expression of HLA-DR on classical, intermediate, and non-classical monocytes also increased after vaccination, showing a similar trend to that of total monocytes (**Figure S1C**). These data suggested that the CoronaVac vaccine likely enhanced the antigen-presenting ability of monocytes effectively.

### Alteration of NK cell frequencies and subsets during CoronaVac vaccination

**Figure 2:**
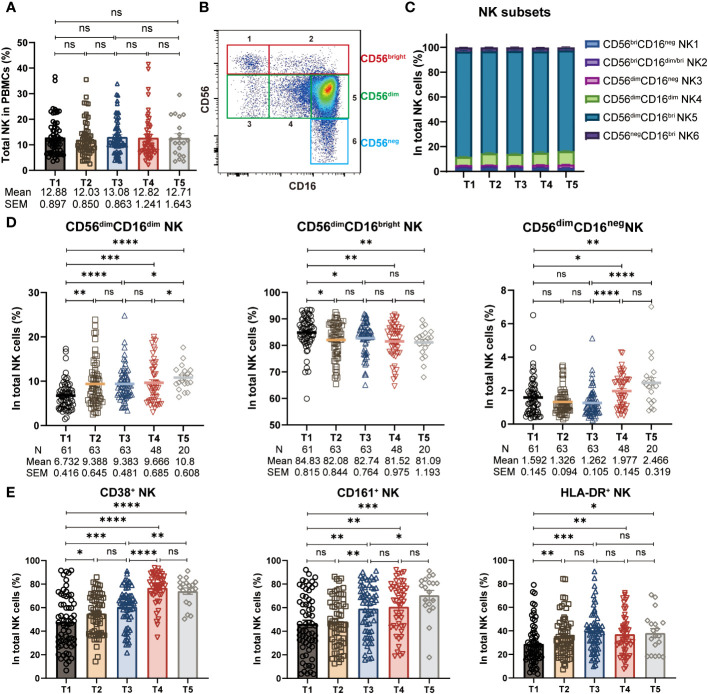
Alteration of NK cell frequencies and subsets during CoronaVac vaccination. **(A)** Statistical analysis of the frequency of polyclonal total NK cells in PBMCs at five time points. **(B)** Representative FACS plots of six NK subsets defined by the density of CD56 and CD16. **(C)** The proportion of six NK subsets (1: CD56^bright^CD16^neg^, 2: CD56^bright^CD16^dim/bright^, 3: CD56^dim^CD16^neg^, 4: CD56^dim^CD16^dim^, 5: CD56^dim^CD16^bright^, 6: CD56^neg^CD16^bright^) in total NK cells at five time points. **(D)** Longitudinal frequencies of polyclonal CD56^dim^CD16^dim^, CD56^dim^CD16^bright^ and CD56^dim^CD16^neg^ NK cells measured by flow cytometry at five time points. **(E)** Statistical analysis of the frequency of CD38^+^, CD161^+^ and HLA-DR^+^ NK cells at five time points. Each dot represents an individual subject. Bars in the figure represent the mean values with SEM. Mann-Whitney U test was applied for comparison between time points **(A, D, E)**. *, *P* < 0.05; **, *P* < 0.01; ***, *P* < 0.001; ****, *P* < 0.0001; ns, not significant.

The proportion of total NK cells in PBMCs remained rather stable throughout the three-dose CoronaVac vaccination regimen, resembling previous findings in mRNA vaccine (24) (**Figure 2A**). NK cells can be further divided into six subsets featured by the differential expression of CD56 and CD16 (16–18): (1) CD56^bright^CD16^neg^; (2) CD56^bright^CD16^dim/bright^; (3) CD56^dim^CD16^neg^; (4) CD56^dim^CD16^dim^; (5) CD56^dim^CD16^bright^; (6) CD56^neg^CD16^bright^ (**Figure S2A**, **Figure 2B**). We thus wondered whether the frequencies of NK cell subsets were altered during the study period. To address this issue, we analyzed the blood NK subsets distribution by multicolor flow cytometry. As expected, CD56^dim^CD16^bright^ NK cells accounted for the majority of total NK cells (**Figure 2C**). However, we discovered the alteration in the proportion of NK cell subsets, resulting in a preferential stimulation of CD56^dim^CD16^dim^ NK cells and CD56^dim^CD16^neg^ NK cells, together with a decreased frequency of CD56^dim^CD16^bright^ NK cells. Specifically, the frequency of CD56^dim^CD16^dim^ NK cells exhibited with a marked increase shortly after the 1^st^ and 2^nd^ dose of vaccination (T2, T3), and with a further increase post 3^rd^ dose of vaccine (T5), as compared to baseline (T1), whereas the frequency of CD56^dim^CD16^bright^ NK cells showed the opposite trend (**Figure 2D**). Additionally, we did not find any significant changes in the frequency of other NK cell subsets (**Figure S2B**). Post 1^st^ and a 2^nd^ dose of CoronaVac, we observed comparable CD56^dim^CD16^neg^ NK cells in vaccinees, while this frequency of CD56^dim^CD16^neg^ NK cells were significantly increased after 6-8 months and maintained at similar level upon a 3^rd^ boost of CoronaVac vaccine (**Figure 2D**). The kinetics and the rapid alterations of CD56^dim^CD16^neg^, CD56^dim^CD16^dim^ NK and CD56^dim^CD16^bright^ NK cells following each vaccine administration support that NK cells are initiated effectively soon after vaccination and may trigger the subsequent vaccine responses. Next, we analyzed the expression of activation markers on NK cells, including CD38, CD161, and HLA-DR. The results showed that compared to baseline (T1), the proportion of NK cells expressing CD38, CD161, and HLA-DR significantly increased after vaccination (**Figure 2E**), indicating that the inactivated CoronaVac vaccine has the ability to induce NK cell activation.

### IFN-γ production by spike-stimulated NK cells

**Figure 3:**
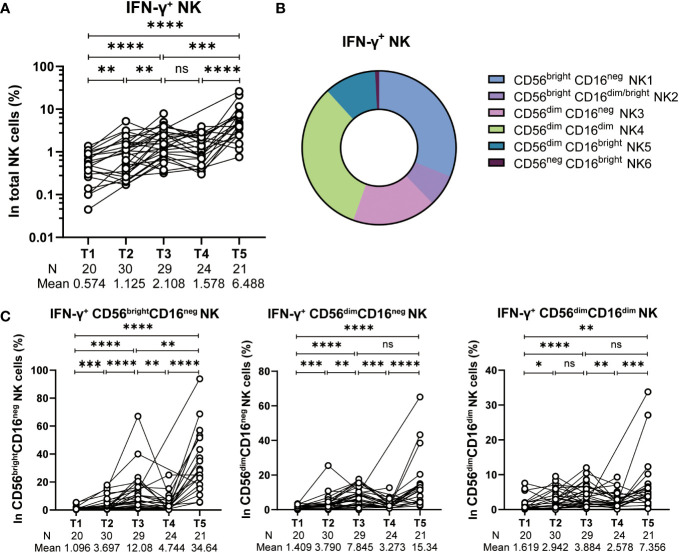
IFN-γ production by spike-stimulated NK cells. **(A)** Statistical analysis of the frequency of IFN-γ production total NK cells stimulated with SARS-CoV-2 spike peptide pools at five time points. **(B)** Subset composition of IFN-γ^+^ NK cells defined by CD16 and CD56. **(C)** Alteration of the frequency of IFN-γ^+^ CD56^bright^CD16^neg^ NK, IFN-γ^+^ CD56^dim^CD16^neg^ NK and IFN-γ^+^ CD56^dim^CD16^dim^ NK cells at five time points. Each black dot represents an individual subject. Statistics were calculated using Wilcoxon matched-pairs signed-ranks test for the comparison between time points **(A, C)** *, *P* < 0.05; **, *P* < 0.01; ***, *P* < 0.001; ****, *P* < 0.0001; ns, not significant.

Several findings support IFN-γ production as the key response that governs neutralizing antibody responses elicited by mRNA vaccines (11, 12, 25). Given that NK cells are both robust and early source of IFN-γ producers during an immune response, we evaluated IFN-γ-producing NK cells after stimulating PBMC with SARS-CoV-2 spike peptides. The frequency of IFN-γ-producing NK cells increased significantly post 1st dose of vaccine (T2), with a further increase post 2^nd^ dose of vaccine (T3). However, 6-8 months after the 2^nd^ vaccine dose (T4), the frequency of IFN-γ-producing NK cells exhibited a deceased trend comparing to that of 2 weeks after the 2^nd^ vaccination (T3). Notwithstanding, the IFN-γ-producing NK cells still retained at a relative high level and higher than that of in the baseline (**Figure 3A**). With that being observed, a 3^rd^ booster of CoronaVac reinvigorated the IFN-γ-producing NK cells efficiently. The average frequency of IFN-γ-producing NK cells reached 6.488 at 2 weeks post dose 3 (T5), a 4.1-fold higher than that at T4 and 11.3-fold higher than that at the baseline T1 (**Figure 3A**). Next, through the analysis of NK cell subpopulations, we further found that CD56^bright^CD16^neg^ NK cells, CD56^dim^CD16^neg^ NK cells and CD56^dim^CD16^dim^ NK cells constitute the main subsets that produce IFN-γ cytokines (**Figure 3B**). The dynamic trends of these three NK cell subsets producing IFN-γ at different time points after CoronaVac vaccination resembled the total IFN-γ^+^ NK cells (**Figure 3C**). Collectively, these results indicate robust induction of IFN-γ-producing NK cells cell responses after vaccination.

## Discussion

Induction of effective adaptive immune responses including the pathogen specific neutralizing antibodies and long-lived memory T cell responses is the main goal of vaccine design, regardless the platforms on which COVID-19 vaccines are based (26). A growing body of evidence now indicating that the innate immune response is critical to the orchestration of protective adaptive immune responses after vaccination (21, 27). It is reported that after vaccination with a single dose of ChAdOx1 nCoV-19, monocytes exhibited enhanced antigen presentation function and cytokine/chemokine profile, which is likely to facilitate adaptive memory responses development (21). In addition, mRNA vaccines can also stimulate modest innate immune responses after primary immunization, while increase notably after the secondary immunization (11, 17, 25). Together, these findings imply that both adenoviral-based and mRNA-based vaccines have heterologous immunological effects on innate responses. However, how would vaccine-induced innate immune response evolve longitudinally has been, and remains, a major unknown for inactivated vaccines. Given that monocytes and NK cells are two main innate cell populations, both of which as important first-line responders to viral infections, we here performed a longitudinal assessment of the monocytes and NK cell responses, elicited by CoronaVac inactivated vaccine in the circulation.

Monocytes can be phenotypically and functionally characterized as classical (CD14^+^/CD16^-^), intermediate (CD14^+^/CD16^+^), and non-classical (CD14^low^/CD16^+^) monocytes (28). They played specific roles in the control and development of immune processes. Classical monocytes participate in tissue repair, inflammatory response, and induce adaptive immune response (29, 30). In contrast, intermediate monocytes have been shown to stimulate T cell proliferation and highly express MHC-II restricted antigen presentation related genes (31, 32). Consistent with the relevant results of the BNT162b1 mRNA vaccine research (17), we found that CoronaVac vaccination significantly increased the frequency of classical and intermediate monocytes. The enhancement of these two subpopulations may imply that vaccination can induce strong antiviral responses and further stimulate adaptive immune responses. According to the report, CD14^+^ monocytes were increased up to 3 months after vaccination with ChAdOx1 nCoV-19 (21). By contrast, the vaccination of three doses of CoronaVac had little effect on the frequency of CD14^+^ monocytes. Next, we further discovered that, consistent with the ChAdOx1 nCoV-19 vaccine (21), the inactivated vaccine also markedly increased the expression level of HLA-DR in CD14^+^ monocytes, indicating that the immune activation and antigen presentation abilities of monocytes were effectively enhanced over a long period of time after CoronaVac vaccination.

NK cells are conventionally subdivided into the two well-characterized functional CD56^bright^CD16^-^ and CD56^dim^CD16^+^ subsets (33). The former subset is considered to be the cytokine-producing NK-cell subset, while the latter subset is considered to be the cytolytic NK-cell subset (33). However, this dichotomy has been ended up with three additional different NK cell populations: CD56^bright^CD16^dim/bright^, CD56^dim^CD16^neg^ and CD56^dim^CD16^dim^ (18). CD56^dim^CD16^dim^ NK subsets with lower maturity were considered as the precursor cells of CD56^dim^CD16^bright^ cells and were functionally in the intermediate position between CD56^dim^CD16^neg^ cells and CD56^dim^CD16^bright^ cells (16).

Understanding the evolution of NK cell subsets during vaccination is helpful to fully understand the immune mechanism of how the inactivated vaccine prevent virus infection. Our results here revealed that the proportion of total NK (CD16^+^ CD56^+^) cell frequencies remained stable at all timepoints, similar to the results observed in BNT162b2 mRNA vaccine (24). However, our data showed an altered composition of NK subpopulations with an increased frequency of CD56^dim^CD16^dim^ NK cells and a decreased frequency of CD56^dim^CD16^bright^ NK cells after the vaccination. The alteration of CD56^dim^CD16^dim^ and CD56^dim^CD16^bright^ NK cells was similar to that reported in BNT162b1 mRNA vaccine (17). A study on COVID-19 convalescent patients revealed that the SARS-CoV-2 antigen peptide pools-activated IFN-γ-producing NK cell counts were correlated with the development of neutralizing antibodies, and that synergistic humoral and cellular immune response could help the effective elimination of the virus (34). In order to further investigate the functional characteristics of CoronaVac-induced CD56^dim^CD16^dim^ NK and CD56^dim^CD16^neg^ NK cells, we detected the IFN-γ produced by NK cell subsets. We found that the frequency of IFN-γ-producing NK cells increased after the 1^st^ and 2^nd^ vaccination, with an even more significantly increase post the 3^rd^ vaccination, indicating that the 3^rd^ booster dose of inactivated vaccine induced an enhanced innate immune response. Due to the fact that CD56^dim^CD16^dim^ NK cells can produce more cytokines compared to CD56^dim^CD16^bright^ NK cells, we thus speculated that CD56^dim^CD16^bright^ NK cells may transform into the CD56^dim^CD16^dim^ NK cells with higher levels of IFN-γ production after CoronaVac vaccination. These data, together, indicates that the three-dose inactivated CoronaVac vaccination elicited robust IFN-γ-producing NK cell response.

This study has some limitations, including the relatively small number of recruited individuals due to the difficulty of long-term longitudinal follow-up. Additionally, we acknowledge that this work was limited in the study of the very early innate immune responses, since the samples were collected 1-2 weeks after each vaccine dose. Nonetheless, our study revealed the alterations in the frequency and function of monocytes and NK cell subpopulations at different time points after inactivated vaccine administration, providing insights for the evaluation of innate immune response during inactivated CoronaVac vaccination. The advantage of this study is that, based on the time points of sample collection, we can dynamically monitor the activation of monocytes and NK cell subsets after each dose of the inactivated vaccine to evaluate the effectiveness, robustness and persistence of innate immune response after vaccination. In general, the results of this study would provide necessary insights on innate immune cells in the context of three dose inactivated CoronaVac vaccination and supply immunological basis for the designment of future vaccines against SARS-CoV-2 and other viruses.

## Data availability statement

The raw data supporting the conclusions of this article will be made available by the authors, without undue reservation.

## Ethics statement

The studies involving humans were approved by the medical ethical committee of the Affiliated Hospital of Jiangnan University (LS2021004). The studies were conducted in accordance with the local legislation and institutional requirements. The participants provided their written informed consent to participate in this study.

## Author contributions

CC: Data curation, Formal Analysis, Software, Writing – original draft, Writing – review & editing. JJ: Methodology, Writing – review & editing. ML: Investigation, Writing – review & editing. YD: Investigation, Writing – review & editing. TC: Writing – review & editing. TJ: Writing – review & editing. FG: Funding acquisition, Project administration, Resources, Supervision, Writing – original draft, Writing – review & editing.
