# Supplementary material for: Longitudinal evaluation of innate immune responses to three doses of CoronaVac vaccine
Source: Front Immunol. 2023 Oct 2;14:1277831. doi: 10.3389/fimmu.2023.1277831 (PMC10577214; doi:10.3389/fimmu.2023.1277831)
Supplement: Supplementary file 1 [file DataSheet_1.docx]

**Supplementary Figure**

**
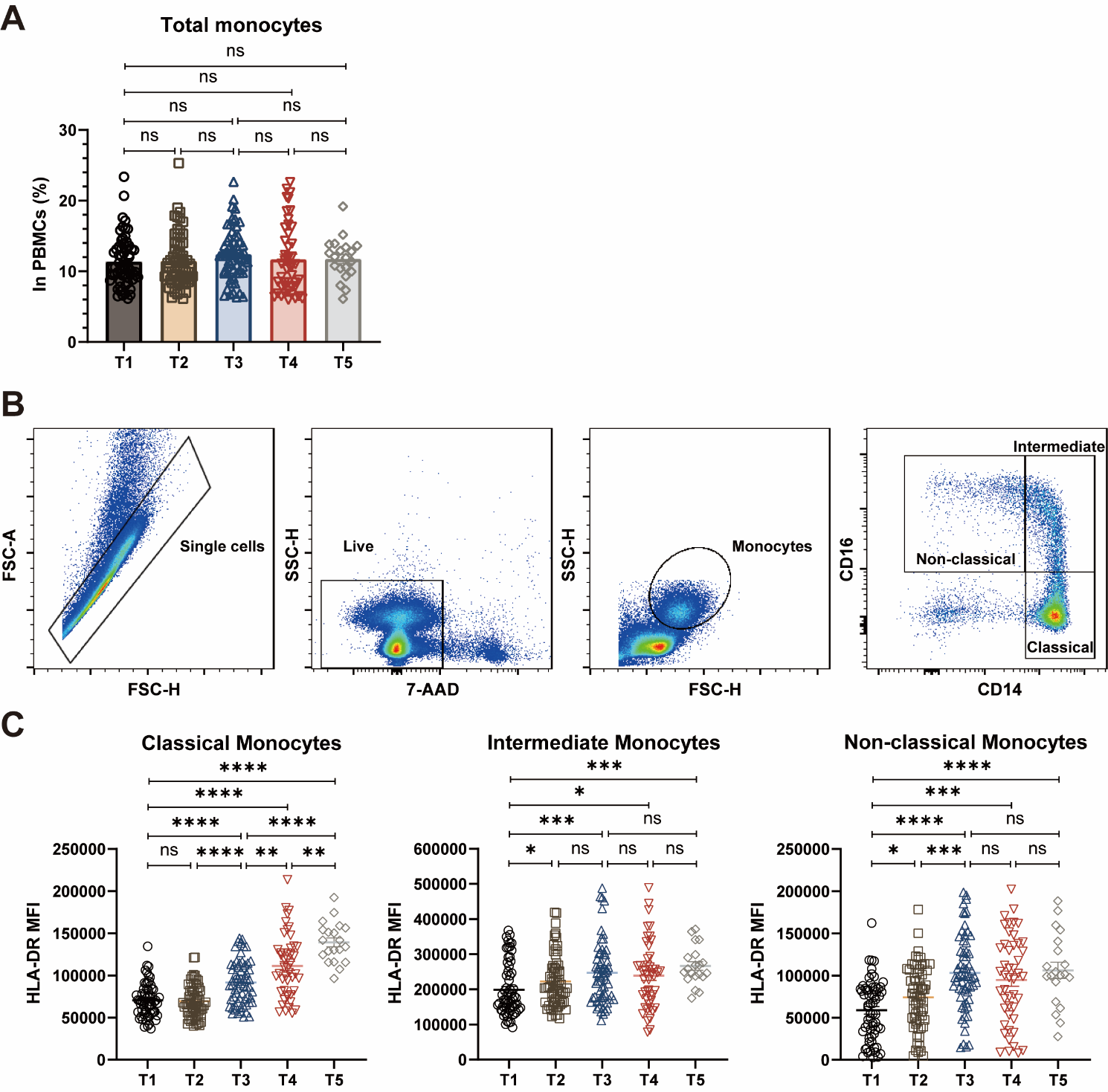
**

**Figure S1: Characterization of monocyte frequencies and subsets during CoronaVac vaccination**

(A) Statistical analysis of the frequency of total monocytes at five time points. (B) Representative plots illustrate the gating strategy of monocytes. Single cells were gated by FSC-H and FSC-A signals. Live cells were identified by 7-AAD, and then monocytes were gated. Monocyte subpopulations can be further identified as CD14^+^CD16^-^ classical monocytes, CD14^+^CD16^+^ intermediate monocytes and CD14^dim^CD16^+^ non-classical monocytes. (C) The median fluorescence intensity (MFI) of HLA-DR expression on classical, intermediate and non-classical monocytes at five time points. Each dot represents an individual subject. Bars represent the mean values with SEM. Mann-Whitney U test was used for comparison between time points (A, C). *, *P* < 0.05; **, *P* < 0.01; ***, *P* < 0.001; ****, *P* < 0.0001; ns, not significant.

**
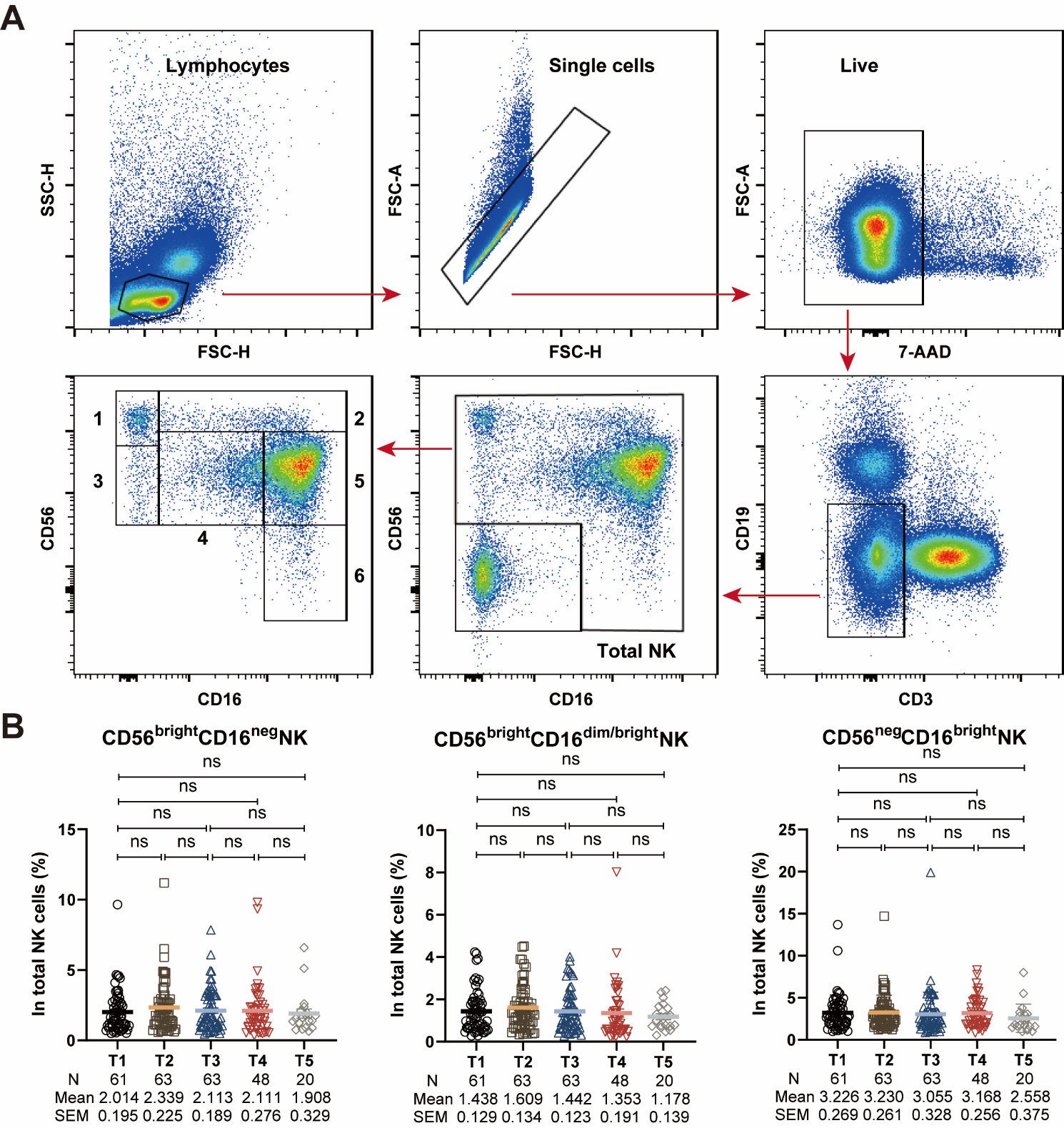
**

**Figure S2: Alteration of NK subsets frequencies during CoronaVac vaccination**

(A) Representative plots illustrate the gating strategy of NK cells and subsets. Lymphocytes were firstly gated, and doublets were excluded by FSC-H and FSC-A signals. Live cells were identified by 7-AAD. Total NK cells were gating from CD3^-^CD19^-^ cells, and defined as CD56^+^CD16^+^ cells. NK cells can be further divided into six subsets based on the expression patterns of CD56 and CD16: (1) CD56^bright^CD16^neg^; (2) CD56^bright^CD16^dim/bright^; (3) CD56^dim^CD16^neg^; (4) CD56^dim^CD16^dim^; (5) CD56^dim^CD16^bright^; (6) CD56^neg^CD16^bright^. (B) Statistical analysis of the frequencies of CD56^bright^CD16^neg^, CD56^bright^CD16^dim/bright^ and CD56^neg^CD16^bright^ NK cell subsets at five time points. Each dot represents an individual subject. Bars represent the mean values with SEM. Mann-Whitney U test was used for comparison between time points (B). ns, not significant.
