# Supplementary material for: Longitudinal evaluation of innate immune responses to three doses of CoronaVac vaccine
Source: Front Immunol. 2023 Oct 2;14:1277831. doi: 10.3389/fimmu.2023.1277831 (PMC10577214; doi:10.3389/fimmu.2023.1277831)
Supplement: Supplementary file 2 [file DataSheet_2.docx]

**Table S1: Basic information and vaccination time of individuals**

| No | Gender | Age | T1 | Dose1 | T2 | Dose2 | T3 | T4 | Dose3 | T5 |
| --- | --- | --- | --- | --- | --- | --- | --- | --- | --- | --- |
| S1 | M | 57 | Day 0 | Day 0 | Day 9 | Day 23 | Day 37 | Day 256 |  |  |
| S2 | F | 46 | Day 0 | Day 0 | Day 7 | Day 14 | Day 28 | Day 252 | Day 316 | Day 330 |
| S3 | F | 46 | Day 0 | Day 0 | Day 7 | Day 14 | Day 28 | Day 252 | Day 316 | Day 330 |
| S4 | M | 43 | Day 0 | Day 0 | Day 7 | Day 14 | Day 28 | Day 255 | Day 311 | Day 325 |
| S5 | F | 32 | Day 0 | Day 0 | Day 7 | Day 14 | Day 28 | Day 252 |  |  |
| S6 | M | 45 | Day 0 | Day 0 | Day 7 | Day 14 | Day 28 | Day 253 | Day 310 | Day 324 |
| S7 | M | 48 | Day 0 | Day 0 | Day 7 | Day 14 | Day 29 | Day 255 |  |  |
| S8 | M | 44 | Day 0 | Day 0 | Day 7 | Day 14 | Day 28 | Day 251 | Day 303 | Day 317 |
| S9 | M | 58 | Day 0 | Day 0 | Day 7 | Day 16 | Day 30 | Day 248 |  |  |
| S10 | F | 34 | Day 0 | Day 0 | Day 7 | Day 16 | Day 30 | Day 245 |  |  |
| S11 | F | 35 | Day 0 | Day 0 | Day 7 | Day 30 | Day 44 |  |  |  |
| S12 | F | 39 | Day 0 | Day 0 | Day 7 | Day 14 | Day 30 | Day 225 | Day 281 | Day 295 |
| S13 | F | 45 | Day 0 | Day 0 | Day 7 | Day 14 | Day 29 | Day 222 | Day 280 | Day 294 |
| S14 | F | 32 | Day 0 | Day 0 | Day 7 | Day 14 | Day 29 | Day 222 | Day 280 | Day 294 |
| S15 | M | 41 | Day 0 | Day 0 | Day 7 | Day 14 | Day 29 | Day 222 |  |  |
| S16 | M | 52 | Day 0 | Day 0 | Day 7 | Day 14 | Day 29 | Day 226 |  |  |
| S17 | F | 50 | Day 0 | Day 0 | Day 7 | Day 14 | Day 29 | Day 224 | Day 274 | Day 288 |
| S18 | F | 48 | Day 0 | Day 0 | Day 7 | Day 14 | Day 29 | Day 222 | Day 275 | Day 289 |
| S19 | M | 39 | Day 0 | Day 0 | Day 7 | Day 15 | Day 30 | Day 226 |  |  |
| S20 | M | 40 | Day 0 | Day 0 | Day 7 | Day 14 | Day 29 | Day 224 |  |  |
| S21 | M | 45 | Day 0 | Day 0 | Day 7 | Day 14 | Day 29 | Day 223 |  |  |
| S22 | M | 43 | Day 0 | Day 0 | Day 7 | Day 14 | Day 29 | Day 223 | Day 275 | Day 289 |
| S23 | M | 42 | Day 0 | Day 0 | Day 7 | Day 14 | Day 29 | Day 224 |  |  |
| S24 | M | 46 | Day 0 | Day 0 | Day 7 | Day 19 | Day 33 | Day 224 |  |  |
| S25 | M | 38 | Day 0 | Day 0 | Day 7 | Day 14 | Day 29 | Day 222 |  |  |
| S26 | F | 44 | Day 0 | Day 0 | Day 7 | Day 14 | Day 30 | Day 223 |  |  |
| S27 | M | 46 | Day 0 | Day 0 | Day 7 | Day 14 | Day 29 | Day 224 | Day 274 | Day 288 |
| S28 | F | 38 | Day 0 | Day 0 | Day 7 | Day 14 | Day 29 | Day 224 | Day 275 | Day 289 |
| S29 | M | 49 | Day 0 | Day 0 | Day 7 | Day 14 | Day 28 | Day 223 |  |  |
| S30 | M | 44 | Day 0 | Day 0 | Day 7 | Day 14 | Day 30 | Day 224 | Day 275 | Day 289 |
| S31 | M | 30 | Day 0 | Day 0 | Day 7 | Day 15 | Day 30 | Day 224 | Day 281 | Day 295 |
| S32 | M | 43 | Day 0 | Day 0 | Day 7 | Day 14 | Day 29 | Day 222 | Day 285 | Day 299 |
| S33 | F | 51 | Day 0 | Day 0 | Day 7 | Day 14 | Day 29 | Day 224 |  |  |
| S34 | M | 45 | Day 0 | Day 0 | Day 7 | Day 14 | Day 29 | Day 221 |  |  |
| S35 | M | 46 | Day 0 | Day 0 | Day 7 | Day 14 | Day 29 | Day 222 | Day 277 | Day 291 |
| S36 | F | 49 | Day 0 | Day 0 | Day 7 | Day 14 | Day 29 | Day 221 | Day 297 | Day 311 |
| S37 | F | 43 | Day 0 | Day 0 | Day 7 | Day 14 | Day 29 | Day 221 |  |  |
| S38 | M | 35 | Day 0 | Day 0 | Day 7 | Day 14 | Day 29 | Day 244 | Day 275 | Day 289 |
| S39 | F | 33 | Day 0 | Day 0 | Day 8 | Day 14 | Day 28 | Day 217 |  |  |
| S40 | F | 28 | Day 0 | Day 0 | Day 8 | Day 14 | Day 28 | Day 217 | Day 277 | Day 291 |
| S41 | M | 54 | Day 0 | Day 0 | Day 8 | Day 48 | Day 62 | Day 239 |  |  |
| S42 | F | 63 | Day 0 | Day 0 | Day 9 | Day 50 | Day 64 |  | Day 225 | Day 239 |
| S43 | M | 64 | Day 0 | Day 0 | Day 15 | Day 44 | Day 58 | Day 231 |  |  |
| S44 | F | 64 | Day 0 | Day 0 | Day 9 | Day 50 | Day 64 |  |  |  |
| S45 | M | 56 | Day 0 | Day 0 | Day 7 | Day 46 | Day 67 | Day 235 |  |  |
| S46 | F | 41 | Day 0 | Day 0 | Day 7 | Day 49 | Day 63 | Day 232 |  |  |
| S47 | F | 38 | Day 0 | Day 0 | Day 7 | Day 48 | Day 62 | Day 231 | Day 239 | Day 253 |
| S48 | F | 56 | Day 0 | Day 0 | Day 7 | Day 51 | Day 65 | Day 231 |  |  |
| S49 | M | 62 | Day 0 | Day 0 | Day 7 | Day 46 | Day 63 | Day 231 |  |  |
| S50 | M | 57 | Day 0 | Day 0 | Day 7 | Day 49 | Day 63 | Day 236 | Day 244 | Day 258 |
| S51 | F | 49 | Day 0 | Day 0 | Day 7 | Day 48 | Day 62 | Day 231 |  |  |
| S52 | F | 37 | Day 0 | Day 0 | Day 7 | Day 67 | Day 81 | Day 250 |  |  |
| S53 | F | 38 | Day 0 | Day 0 | Day 7 | Day 49 | Day 63 | Day 235 |  |  |
| S54 | F | 59 | Day 0 | Day 0 | Day 7 | Day 56 | Day 70 | Day 244 |  |  |
| S55 | F | 59 | Day 0 | Day 0 | Day 7 | Day 56 | Day 70 |  |  |  |
| S56 | F | 50 | Day 0 | Day 0 | Day 10 | Day 49 | Day 63 | Day 231 | Day 232 | Day 246 |
| S57 | M | 61 | Day 0 | Day 0 | Day 7 | Day 64 | Day 77 | Day 239 |  |  |
| S58 | M | 57 | Day 0 | Day 0 | Day 8 | Day 63 | Day 77 | Day 240 |  |  |
| S59 | F | 67 | Day 0 | Day 0 | Day 7 | Day 49 | Day 65 | Day 232 |  |  |
| S60 | F | 66 | Day 0 | Day 0 | Day 7 | Day 51 | Day 65 | Day 232 |  |  |
| S61 | M | 51 | Day 0 | Day 0 | Day 12 | Day 45 | Day 59 | Day 234 | Day 248 | Day 262 |
| S62 | M | 51 | Day 0 | Day 0 | Day 7 | Day 46 | Day 60 | Day 230 | Day 238 | Day 252 |

Note: F, Female, M, Male. T1: pre-vaccination baseline, T2: 1 week post the primary dose, T3: 2 weeks post the 2^nd^ dose, T4: 6-8 months post the 2^nd^ dose, T5: 2 weeks post the 3rd booster dose.

**Table S2: Antibodies and other key resources**

| **REAGENT or RESOURCE** | **SOURCE** | **IDENTIFIER** |
| --- | --- | --- |
| **Antibodies** | | |
| Alexa Fluor 532 Hu CD3 (Clone: UCHT1) | eBioscience | Cat# 58-0038-42 |
| PE-Cy5 Anti-Human CD14 (Clone: 61D3) | Thermo | Cat# 15-0149-42 |
| APC/Fire 750 Anti-Human HLA-DR (Clone: L243) | BioLegend | Cat# 307658 |
| Super Bright 436 Anti-Human CD19 (Clone: HIB19) | eBioscience | Cat# 62-0199-42 |
| eFluor 450 Anti-Human CD16 (Clone: CB16) | eBioscience | Cat# 48-0168-42 |
| Brilliant Violet 711 Anti-Human CD56 (NCAM) (Clone: HCD56) | BioLegend | Cat# 318336 |
| PE-Cy7 Anti-Human IFN-γ (Clone: 4S.B3) | BioLegend | Cat# 502528 |
| PerCP-eFluor 710 Anti-Human CD38 (Clone: HB7) | eBioscience | Cat# 46-0388-42 |
| Brilliant Violet 785 Anti-Human CD161 (Clone: HP-3G10) | BioLegend | Cat# 339930 |
| **Chemicals, Peptides, and Recombinant Proteins** | | |
| 7AAD Staining Solution | eBioscience | Cat# 00-6993-50 |
| FACS Lysing Solution | BD Pharmingen | Cat#349202 |
| Human BD Fc Block | Miltenyi | Cat#130-059-901 |
| Ficoll-Paque^TM^ PLUS Media | GE Healthcare Life Sciences | Cat#17144002 |
| FBS | Gibco | Cat#10100147 |
| SARS-CoV-2 spike Peptide Pool | SinoBiological | Cat#PP003 |
| Cytofix/Cytoperm | BD Pharmingen | Cat#51-2090KZ |
| BD Perm/Wash | BD Pharmingen | Cat#51-2091KZ |
